# Marine Skeletal Biopolymers and Proteins and Their Biomedical Application

**DOI:** 10.3390/md19070389

**Published:** 2021-07-12

**Authors:** M. Azizur Rahman

**Affiliations:** 1 Centre for Climate Change Research, Toronto, ON M4P 1J4, Canada; aziz@climatechangeresearch.ca or mazizur.rahman@utoronto.ca; 2 A.R. Environmental Solutions, ICUBE-University of Toronto Mississauga, Toronto, ON L5L 1C6, Canada

Skeletal biopolymers and proteins in marine organisms are present as complex mixtures and have great potential applications in the biomedical field. The organic matrices of marine calcifiers are the main source of skeletal proteins [1,2,3,4,5,6] and they have very interesting structural formation. Marine skeletal proteins are also a very rich source of amino acids, which are essential for building good health. Similarly, biopolymers from marine resources have a variety of structural characteristics that make them useful for different biomedical applications. However, due to their broad array of biological functions in biopolymer- and protein-based drugs, such as anticancer, antimicrobial, bone tissue regeneration, antioxidant, and anti-aging activities, bioactive skeletal proteins and biopolymer have recently attracted a great amount of interest in the pharmaceutical, nutraceutical, and cosmeceutical industries.

The advantages of marine sources are their huge availability and abundance in the shallow, mid-level, and deep-sea waters. These sources include marine invertebrates and related calcifiers, for example, soft and hard corals, mollusks/bivalves, sponges, sea urchins, coralline red algae, and other calcifying marine organisms [7,8,9,10,11,12,13,14].

This Special Issue of Marine Drugs on marine skeletal biopolymers and proteins contains 13 high-quality original articles on different interesting topics related to biomedical and other applications. In the following sections, a short overview of the research findings contributed by the authors is provided, which could help readers to find their relevant articles.

Zheng et al. investigated the antioxidant activity of SNNH-1 in vitro and their findings showed that SNNH-1 can be used as a marine antioxidant and provide a basis for its application in the food and pharmaceutical fields. Five different proteases were used to hydrolyze the swim bladders of *Nibea japonica*, and the hydrolysate treated with neutrase (collagen peptide named SNNHs) showed the highest scavenge 2, 2-diphenyl-1-picrylhydrazyl (DPPH) radical scavenging activity. These results imply that collagen peptides from *Nibea japonica* can significantly reduce the oxidative stress caused by H_2_O_2_, providing a basis for the application of collagen peptides in the food industry, pharmaceuticals, and cosmetics [15].

Benayahu et al. analyzed the biocompatibility of a marine collagen-based scaffold applied both in vitro and in vivo in a rat animal model [16]. The experiment demonstrated the healing of a rotator cuff tear, the most common musculoskeletal injury occurring in the shoulder. The designed biomaterial will allow the future development of bio composite-based products with optimal mechanical properties that will fully integrate with the natural tissue, contributing to its healing processes. This study also demonstrated that the 3D structure facilitates cell migration and new blood vessel formation needed for tissue repair.

The application of sponges for water purification and collagen production was demonstrated by Gökalp et al. [17]. The main goal of this study was to investigate the effect of depth on the filtration capacity (measured as in situ bacterial clearance rates), metabolism (respiration rate as oxygen consumption), morphology (density and size of oscula), growth, and collagen/biomass production of *Chondrosia* *reniformis*. This study represents an important step forward, both in understanding the morphological plasticity and performance of sponges and in the application of *C. reniformis* for combined bioremediation and collagen production.

Machalowski et al. investigated chitin-based 3D scaffolds which were isolated from the cultivated under the farming *Aplysina aerophoba* marine demosponge. This biomaterial was modified by silver nanoparticle deposition using chemical reduction of silver nitrate and the antibacterial action was investigated. The results were used for the first time as a basic construct for the fabrication of an antibacterial water filter. This group of marine sponges represents a unique, renewable source of specialized chitin due to their ability to grow under marine farming conditions and which could have a high industrial potential [18].

Hermann Ehrlich and his group, Nowacki et al. [8], reported the isolation technique of chitin from the skeleton of black coral *Cirrhipathes* sp. (Antipatharia, Antipathidae) for the first time. In this study, the authors report the stepwise isolation and identification of chitin from this species and this novel method allows the isolation of α-chitin in the form of a microporous membrane-like material. Additionally, the extracted chitinous scaffold, with a well-preserved, unique pore distribution, was extracted in an amazingly short time (12 h).

Zhang et al. explored the structure of pepsin-solubilized collagen (PSC) from the skin of *Lophius litulon*. The authors used a variety of techniques such as sodium dodecylsulphate polyacrylamide gel electrophoresis (SDS-PAGE), Fourier transform infrared spectroscopy (FTIR), and scanning electron microscopy (SEM). The protein analysis results by SDS-PAGE revealed that PSC from *Lophius litulon* skin was collagen type I and had collagen-specific α1, α2, β, and γ chains. The overall results suggest that collagen from the skin of *Lophius litulon* has potential applications in wound healing along with physicochemical and antioxidant properties due to its good biocompatibility [19].

In the article by Gaspar-Pintiliescu [20], gelatin from the soft tissue of *Rapana venosa* was extracted and characterized using acidic and enzymatic methods. The main purpose of this study was to use the results in the pharmaceutical and cosmetic fields. Its physicochemical and ultrastructural properties were analyzed and compared to those of commercial pig skin gelatin. In addition, gelatins were tested on human keratinocyte cells for their cytocompatibility, cell adhesion capacity, and irritant potential.

Kovalchuk et al. published an interesting work entitled “Naturally Drug-Loaded Chitin: Isolation and Applications”. In this study, the authors report the demosponge *Ianthella flabelliformis* (Linnaeus, 1759) for concurrent extraction of both naturally occurring (“ready-to-use”) chitin scaffolds, and biologically active bromotyrosines, which are recognized as a potential antibiotic, antitumor, and marine antifouling substances. The results demonstrated that sponge-derived chitin scaffolds, impregnated with decamethoxine, effectively inhibit the growth of human pathogen *Staphylococcus aureus* in an agar diffusion assay [21].

Chen et al. reported the presence of type 1 collagen in red stingray skin. In this study, the authors extracted acid-soluble collagen (ASC) and pepsin-soluble collagen (PSC) from the skin of red stingray *Dasyatis akajei*, and subsequently, its physicochemical and functional properties were investigated. The authors suggested that the PSC from red stingray skin could be useful (instead of terrestrial animal collagen) in drugs, foods, cosmetics, and biological functional materials, and as scaffolds for bone regeneration [22].

The article by Jin et al. reports on the kinetic resolution of racemic styrene oxide (SO) and benzyl glycidyl ether (BGE) obtained by the variant of epoxide hydrolase from *Agromyces mediolanus* (vEH-Am). This study describes the theoretical foundation for the application of vEH-Am in the preparation of enantiopure SO and BGE [23].

The article by Chen et al. evaluated the in vitro anti-proliferative mechanism between Nereis Active Protease (NAP) and human lung cancer H1299 cells. Their experimental results indicate that colony formation and migration of cells were significantly lowered following NAP treatment. Flow cytometry results suggested that NAP-induced growth inhibition of H1299 cells is linked to apoptosis, and that NAP can arrest the cells at the G0/G1 phase [24]. 

Pan et al. report four antioxidant peptides (RSHP-A, RSHP-B, RSHP-C, and RSHP-D) from protein hydrolysate of red stingray (*Dasyatis akajei*) cartilages. In the work, water-soluble proteins of red stingray (*Dasyatis akajei*) cartilages were extracted by guanidine hydrochloride and hydrolyzed using trypsin [25].

The article by Lin et al. reports the extraction of collagen from bigeye tuna (*Thunnus obesus*) skins by salting-out (PSC-SO) and isoelectric precipitation (PSC-IP) methods. Their results suggest that PSC-IP could be used to rapidly extract collagen from marine by-products instead of traditional salting-out methods. Moreover, collagen from bigeye tuna skin may have strong potential for cosmetic and biomedical applications [26].

As a guest editor, I appreciate the endeavors provided by all of the authors who contributed their excellent results to this Special Issue. I would also like to thank all of the reviewers who carefully evaluated the submitted manuscripts and the editorial board of Marine Drugs for their support and kind help.
